# The Nature and Affinities of Certain Submerged Fungoid Growths

**Published:** 1882-05

**Authors:** E. B. Stuart

**Affiliations:** Pharmaceutical Chemist and Microscopical Expert, Associate Editor of *The Druggist*


### ARTICLE IV.

### THE NATURE AND AFFINITIES OF CERTAIN SUBMERGED FUNGOID GROWTHS. By E. B. Stuart, Pharmaceutical Chemist and Microscopical Expert, Associate Editor of *The Druggist.*

(Read before the Biological Society, April 5, 1882.)

The natural history of some of the lower fungi is a matter of interest to the physician, both from the relation which these plants bear to certain diseases, and from a purely biological aspect.

The whole order of fungi occupies an intermediate ground between plants and animals, and presents here an affinity to one and there to the other grand division of living forms. Among the more highly organized fungi, as the mushroom tribe, the vegetable element predominates in a marked degree; the puff-ball fungus affords a parallel example, with which every one is familiar. Not so with the myseomycetes, some of which in their mature state, resemble a puff-ball so closely as to require the skill of a specialist to distinguish one from the other.

During the vegetative period, however, these plants have such marked animal characteristics, that one of the most eminent authorities in this department of study, once proposed separating this order from the fungi, and transferring it to the animal kingdom. As just stated, the myseomycetes, during their entire period of assimilation and growth, exhibit all the characters of animal life. They are simple masses of protoplasm, which remain perfectly free and exhibit amœboid movements, apparently by volition. The organism retreats from strong light, and shuns dry or metallic surfaces; it spreads itself out in a network of living threads, contracts into a ball of tremulous jelly, or retracts its substance into the crevices of the decaying wood on which it lives.

The motile spores of many fungi have, when superficially con sidered, some characters more animal than plant-like. I refer to their active movements when set free, and the apparent sagacity displayed in seeking their future home. Still this is hardly more wonderful or animal-like than the persistent and systematic manner in which, according to Darwin, the tendrils of climbing plants search for some object to grasp.

The relation of certain fungi to disease is too well-known to the gentlemen of this Society to require mention at my hands.

The immediate subject of this paper, the consideration of submerged fungi, is a minor portion, a fragment, so to speak, of the whole. My attention was attracted to the subject some years ago by the frequent and unwelcome appearance of floculent masses in solutions of the medicinal alkaloids.

Under an inch objective, these masses have the appearance of cottony tufts, made up of white, shining threads of extreme delicacy; more highly magnified, the threads are perceived to be tubular, and, in many cases, partitioned off into long cells, which may contain ordinary granular protoplasm, or in some instances minute spherical bodies, of high refractive power, ranged along like beads, in single file. If these bodies are kept under observation, it will be noticed that the cell which contains them finally disintegrates, and they are set free. After this occurs, provided that the liquid in which they rest is of the proper temperature, and contains a supply of suitable food, the bodies in question begin to bulge a little on one side. This continues until it becomes a prolongation, and the prolongation a thread. The group of pseudo-spores has now become the nucleus of a new growth, precisely like the first, and like the specimen before you.

(Here the lecturer exhibited various specimens, at different stages of growth, under the microscope.)

If any doubt exists as to the vegetable nature of this growth, suitable tests may be applied. It will be found undoubtedly vegetable in its composition. If, however, an attempt is made to locate the plant botanically, the effort will be fruitless. But one small order of eight or ten species among the fungi is aquatic, and the members of this order are all parasitic upon animal substances, and the method of fructification in that order is altogether different from that just described. The spores in the aquatic fungi are developed in well-marked sacs or asci, while these are formed in any portion of the filament indifferently, or, at least, apparently so, for there are no indications of a sac or difference of structure to suggest a special function for this portion of the filament. Then the spores of the saproligniæ, or aquatic fungi, are motile. As soon as set free, they swim actively about, while these pseudo-spores are as motionless as the seeds of the narcotic poppy. The plant corresponds to none of the described fungi, and it certainly does not belong to the algæ, for it contains no chlorophyll, and grows in the dark as well as in the light. The specimens on the table grew in the box which now contains them, and the box has been kept in a drawer of my desk. No chlorophyll-bearing plant would thrive for any length of time under such conditions. If a specimen is transferred from one of these phials to pure water, growth ceases and it soon disintegrates and disappears. If, on the other hand, it is brought into Pasteur's solution, or Mayer's pepsin solution, it grows rapidly until the liquid is quite filled with the plant, and assumes the appearance of a doughy mass.

Now, the experiment may be varied somewhat, and instead of immersing the plant in Mayer's fluid, it may be supplied with a sufficient quantity of this or that suitable food in an ordinary growing cell, or one of Tyndall's culture boxes.

Under these conditions the mass vegetates luxuriantly for a time, and finally sends up delicate hyptræ, bearing its fruits. This affords the means of identification, and in most cases the plant will be found to be some one of the five or six most common molds.

If you brush a camel's hair pencil lightly over a mass of common blue mold and then rinse it in a solution of morphia or strychnia, or, in fact, in almost any of the common medicinal alkaloids, and allow the liquid to stand quietly, at the ordinary living temperature of your rooms, for a few days, these fungoid growths will make their appearance, and may be cultivated for years without ever assuming their normal condition or bearing their proper fruit. This mode of vegetation seem quite analagous to the growth of a perennial plant, which, under favorable conditions of soil and climate, will continue to vegetate for many years without bearing fruit. On removing our submerged pericillium from the liquid in which it was growing under abnormal and unfavorable conditions, it soon resumes its normal habit and bears its proper fruit. In the course of my experiments with these fungi I employed the spores of a great variety of species and varied the conditions of growth as widely as my ingenuity suggested methods. In one or two instances I was able to keep the same plant in this submerged condition for more than a year, and finally to secure its normal fruit on restoring it to the air. The experiments were conducted with reasonable care to guard against infection from spores floating in the air, and the results may, I think, be accepted as indicating strongly the true nature of the growth and its relation to other fungi. Two practical questions hinge upon these experiments, namely: The effect of the growth of the plant upon an alkaloidal solution, and the best means of preventing their growth. In regard to the first point, it is quite clear that any increase in the weight of the plant, beyond that of the spores originally added to the solution, must, if the water used was free from organic matter, have been derived from and at the expense of the alkaloid itself.

While a chlorophyll-bearing plant, which feeds mainly from the acriform matter around us, will grow in a pot of earth without taking anything like its own weight of matter from the soil, this is not the case with fungi; they are essentially parasites, and, like animals, derive their carbonaceous food from the mineral kingdom at second hand; they are utterly incapable of reducing carbon dioxide from the air, and under the circumstances before us, must break up and decompose the alkaloid in which they grow and appropriate it to the formation of their own tissues. It is well known that certain fungi are able to decompose comparatively stable and simple compounds, as sugar, urea, etc., etc., to build up from the material so obtained more complex and less stable substances. Some of these reactions are of commercial importance, and have been very carefully and thoroughly studied, and the nature of the decomposition and the composition of the products are well known. In the case of the medicinal alkaloids, however, the nature of the decomposition of the products formed is, I believe, entirely unknown, and while the changes are, in a general way, undoubtedly analagous to the decomposition of sugar by yeast, we know nothing of the physiological effects of these decomposition products upon the animal organism.

The growth of these fungi may, of course, be prevented by any of the ordinary antiseptics. In solutions for hypodermic use, as small a quantity as 5 per cent. of common alcohol will entirely prevent them. They will seldom make their appearance in solution made with the aromatic waters, as peppermint or dill water. If, in addition to this, the solution is warmed to about 150°, when otherwise completed, and reasonable care be exercised in keeping the container closed, no apprehension of their growth need be felt. For the use of pharmacists, who find these solutions convenient for the dispensing counter, this little contrivance (both with cotton filter and small accessory tube through the cork), the idea of which, you will notice, is that of Pasteur's cotton filter, is useful. The solution is filtered into the bottle, the cork carrying the two tubes inserted, and the whole affair placed in the water bath and heated sufficiently to kill any spores that may be present, the exit tube, bent at a right angle, has a sufficient length to intercept all spores on their inward course, and the funnel tube, which supplies air as the liquid flows out, is stuffed with absorbent cotton, which effectually stops the passage of spores through that tube and preserves the liquid from infection. (See discussion of the paper in Society Reports.)

